# Reconsidering the terminology of “early” and “advanced” gastric cancer: toward a harmonized global lexicon

**DOI:** 10.1007/s10120-026-01717-y

**Published:** 2026-02-07

**Authors:** Takeshi Sano, Narikazu Boku, Florian Lordick

**Affiliations:** 1 Cancer Institute Hospital of the Japanese Foundation for Cancer Research, 3-8-31 Ariake, Kotoku, Tokyo, 135-8550 Japan; 2 Department of Oncology and General Medicine, the IMSUT Hospital, the Institute of Medical Science, the University of Tokyo, Tokyo, Japan; 3 Department of Medicine and University Cancer Center Leipzig, University of Leipzig Medical Center, Leipzig, Germany

**Keywords:** Early gastric cancer, Locally advanced gastric cancer, Advanced gastric cancer

## Abstract

The term *early gastric cancer (EGC)*, defined as carcinoma confined to the mucosa or submucosa irrespective of lymph node status, has been widely accepted worldwide. In contrast, the terminology surrounding *advanced gastric cancer* remains inconsistent across regions. In Western oncology, “advanced cancer” often denotes unresectable or metastatic disease, whereas “early-stage cancer” may include resectable stage II–III tumors. This semantic discrepancy has led to confusion in international communication, clinical trials, and guideline interpretation. We propose a three-tier terminology—*early gastric cancer*, *locally advanced gastric cancer*, and *advanced gastric cancer*—to align the Japanese pathological concept with global clinical staging practice and therapeutic intent.

## Historical background

The concept of *early gastric cancer (EGC)* was first introduced in Japan in the 1960s by gastroenterologists, surgeons, and pathologists, in parallel with major advances in endoscopic diagnosis and surgical pathology [1]. EGC was defined as carcinoma confined to the mucosa or submucosa, irrespective of lymph node metastasis, and this definition has since become established both in Japan and internationally [2]. The widespread adoption of EGC reflects its clinical utility in identifying tumors with an excellent prognosis when treated appropriately.

While the definition of EGC has been clearly articulated and consistently applied, the corresponding terminology for more deeply invasive gastric cancer has not been formally standardized, particularly in English-language usage.

## Semantic discrepancy and clinical confusion

In Japan, gastric cancers that do not meet the definition of EGC are referred to in clinical practice as “advanced gastric cancer,” primarily based on depth of invasion (T2 or deeper) [3]. However, in Western oncology, the term *advanced cancer* is commonly reserved for disease that is unresectable and/or metastatic, typically corresponding to stage IV [4].

Conversely, the term *early-stage cancer* in Western usage frequently encompasses a broader range of disease, including resectable stage II and even stage III tumors [5]. As a result, a gastric cancer described as “advanced” in the Japanese context may be interpreted by Western clinicians as terminal or palliative disease, despite being potentially curable by surgery even without chemotherapy. This discrepancy has practical implications for international collaboration, trial design, and interpretation of outcomes.

## A proposal for harmonization

To address this inconsistency, we propose a three-tier terminology for gastric cancer (Table 1). The term *locally advanced cancer* is already widely used across many solid tumors to describe stage II–III disease that is locally extensive but potentially amenable to curative-intent treatment, often in combination with multimodal therapy.

**Table 1:** Proposal of a three-tier terminology

Term	Definition
*Early gastric cancer*	Tumor confined to the mucosa or submucosa (T1), irrespective of lymph node status
*Locally advanced gastric cancer*	Resectable T2–T4 tumors without distant metastasis, broadly corresponding to stage II–III
*Advanced gastric cancer*	Unresectable and/or metastatic disease, largely corresponding to stage IV

## Tumor-specific considerations of “locally advanced”

It is important to acknowledge that the meaning of *locally advanced cancer* is not uniform across all malignancies. In pancreatic cancer, for example, the term frequently refers to tumors with extensive local vascular involvement that precludes surgical resection despite the absence of distant metastasis [6]. This usage reflects tumor-specific anatomical and biological constraints rather than a general staging principle.

In gastric cancer, however, *locally advanced* has long been used in Western and global clinical trials and guidelines, albeit without a universally accepted formal definition, to describe resectable disease requiring perioperative or neoadjuvant therapy [7–9]. Making this implicit usage explicit by defining *locally advanced gastric cancer* as resectable stage II–III disease may help reduce semantic misunderstanding between regions and facilitate clearer international communication.

## Conclusion

While the concept of *early gastric cancer* has achieved worldwide acceptance, the terminology used to describe more advanced disease has diverged across regions. The unqualified use of “advanced gastric cancer” risks semantic misunderstanding when applied to resectable tumors. We propose adopting a three-tier classification—*early*, *locally advanced*, and *advanced gastric cancer*—to harmonize Japanese pathological concepts with international clinical staging and treatment paradigms. Such a shared lexicon will facilitate clearer communication and stronger global collaboration in gastric cancer care.
